# Engineered clinical-grade mesenchymal stromal cells combating SARS-CoV-2 omicron variants by secreting effective neutralizing antibodies

**DOI:** 10.1186/s13578-023-01099-z

**Published:** 2023-08-31

**Authors:** Yanning Wang, Tianyun Gao, WanTing Li, Chenxu Tai, Yuanyuan Xie, Dong Chen, Shuo Liu, Feifei Huang, Wenqing Wang, Yuxin Chen, Bin Wang

**Affiliations:** 1 Clinical Stem Cell Center, Nanjing Drum Tower Hospital, The Affiliated Hospital of Nanjing Medical School, Nanjing University, 321 Zhongshan Road, Nanjing, 210000 China; 2 Department of Infectious Diseases, Nanjing Drum Tower Hospital Clinical College of Xuzhou Medical University, Nanjing, 21000 China; 3 Department of Laboratory Medicine, Nanjing Drum Tower Hospital Clinical College of Jiangsu University, Nanjing, 21000 China

**Keywords:** MSC, COVID-19, Anti-SARS-CoV-2 antibodies, Gene modification, mAbs delivery platform

## Abstract

**Background:**

The emergence of SARS-CoV-2 becomes life-threatening for the older and immunocompromised individuals, whereas limited treatment is available on these populations. Mesenchymal stromal cells (MSCs) have been reported to be useful in SARS-CoV-2 treatment and reduce SARS-CoV-2-related sequelae.

**Results:**

In this study, we developed an autonomous cellular machine to secret neutralizing antibody in vivo constantly based on the clinical-grade MSCs, to combat SARS-CoV-2 infections. First, various modified recombinant plasmids were constructed and transfected into clinical-grade MSCs by electroporation, for assembly and expression of neutralizing anti-SARS-CoV-2 antibodies. Second, the stable antibody secreting MSCs clones were screened through pseudovirus neutralization assay. Finally, we investigated the pharmacokinetics and biodistribution of neutralizing antibody secreted by engineered MSCs in vivo. The stable clinical-grade MSCs clones, expressing XGv347-10 and LY-CoV1404-5 neutralizing antibodies, exhibited their feasibility and protective efficacy against SARS-CoV-2 infection. Transplanted engineered clinical-grade MSCs effectively delivered the SARS-CoV-2 antibodies to the lung, and the immune hyperresponsiveness caused by COVID-19 was coordinated by MSC clones through inhibiting the differentiation of CD4 + T cells into Th1 and Th17 subpopulations.

**Conclusions:**

Our data suggested that engineered clinical-grade MSCs secreting effective neutralizing antibodies as cellular production machines had the potential to combat SARS-CoV-2 infection, which provided a new avenue for effectively treating the older and immunocompromised COVID-19 patients.

**Supplementary Information:**

The online version contains supplementary material available at 10.1186/s13578-023-01099-z.

## Background

Current severe acute respiratory syndrome coronavirus 2 (SARS-CoV-2) Omicron subvariants are responsible for the ongoing pandemic of COVID-19, which has imposed a heavy burden on global health and caused the deaths of millions of individuals worldwide (https://covid19.who.int). International data have shown that three-dose vaccination continue to be effective to neutralize the Omicron variant and reduce the frequency of severe outcomes [1, 2]. However, vaccination coverage remains low in older individuals (> 65 years old) due to vaccination restriction of some underlying diseases, and older people have high risk of experiencing severe or long-term symptoms associated with omicron variants, and require hospitalization, with increased rates of fatality [3–5]. Thus, developing effective treatment for severe cases, especially the elderly is of global importance.

Many studies have illustrated the clinical safety and efficacy of monoclonal antibodies (mAbs) therapies for COVID-19 patients. It is worth noting that the main target populations for such antibody therapy include those aged over 65 with comorbidities and immunocompromised individuals. The mAb-based approaches can reduce the severity and mortality of these populations [6–8]. To date, several mAb therapies have been applied in clinical practice, such as bamlanivimab (LY-CoV555) and etesevimab [9]. However, new Omicron subvariants BA.2 and BA.4/5 have become dominant worldwide. These new subvariants carrying further mutations raise concerns that they may further evade mAbs. Currently, only bebtelovimab (LY-CoV1404) can still be successfully used in the fight against the Omicron [10].

It is noteworthy that a recent study revealed that antibodies elicited by vaccination had greater binding breadth than antibodies elicited by natural infection, which means that SARS-CoV-2 mutations have less impact on vaccine-elicited antibodies [11].

Over the past decades, mesenchymal stromal cell (MSC) therapies have progressed from a skeptical idea to clinical reality. The safety and efficacy of MSC therapies have been demonstrated in a variety of clinical trials, such as acute respiratory distress syndrome (ARDS) and immune-mediated inflammatory diseases [12–15]. Notably, several clinical data have shown that MSCs therapies can improve clinical outcomes of COVID-19 patients [16, 17]. MSCs can regulate the inflammatory environment and reduce the occurrence of “cytokine storm”, which were the major causes of organ damage that lead to the progression of severe COVID-19 [18]. In addition, MSCs have been used as a suitable gene therapy vectors for in vivo delivery of any therapeutic molecules, such as human soluble tumor necrosis factor receptor II (hsTNFR), interleukin-10 (IL10), and basic fibroblast growth factor (BFGF), demonstrating a promising therapeutic efficacy of genetically engineered MSCs in rheumatoid arthritis (RA) and spinal cord injury (SCI) [19–21]. To date, engineered MSCs that encode various mAbs have shown improved therapeutic efficacy and kinetics compared with direct application of the same antibody [22, 23]. However, no study examines the use of engineered MSCs as autonomous cellular machines for the SARS-CoV-2-specific neutralizing antibodies production to combat SARS-CoV-2 infection. Therefore, we posit that the utility of engineered MSCs as the SARS-CoV-2 neutralizing mAbs delivery platform could substantially improve the clinical outcomes of vulnerable populations by modulating the COVID-19 related immune responses and reducing SARS-CoV-2 entry and replication in vivo.

In this study, we constructed various recombinant plasmids encoding corresponding SARS-CoV-2 antibodies (infection-elicited antibodies 2–15 and LY-CoV1404 as well as vaccine-elicited antibody XGv347) and electroporated these plasmids into clinical-grade MSCs to investigate their feasibility and efficacy to treat SARS-CoV-2 infection. We successfully screened two stable engineered MSCs clones secreting specific mAbs against Omicron subvariants, revealed by pseudovirus neutralization assay. Our study indicated the engineered MSCs as neutralizing mAbs delivery platform could coordinate immune hyperresponsiveness and directly neutralize virus in acute SARS-CoV-2 patients, providing a robust basis for the older or vulnerable individuals with COVID-19.

## Results

### Engineered human clinical-grade MSCs expressing SARS-CoV-2-neutralizing mAbs

**Fig. 1:**
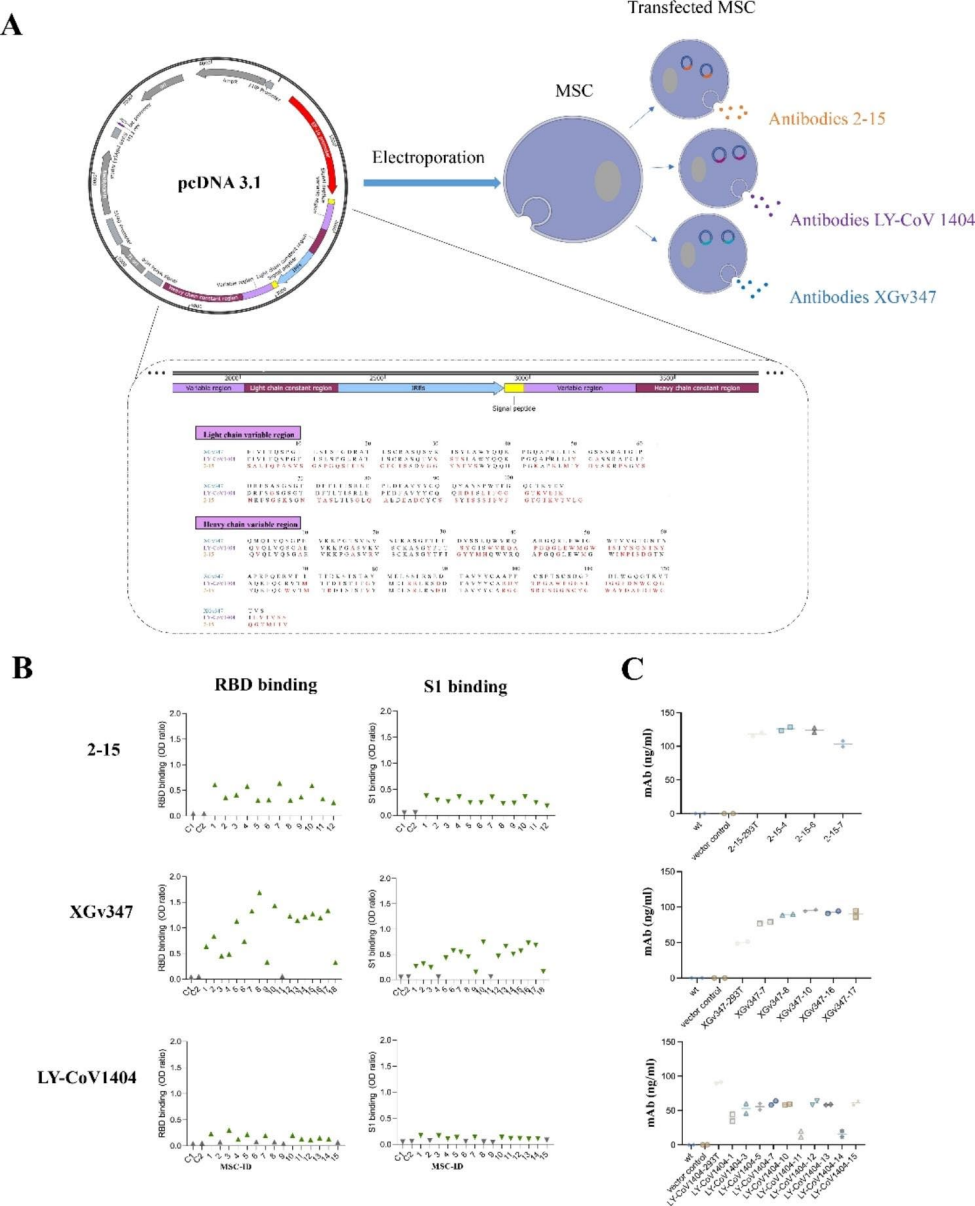
Generation of genetic-modified MSCs secreting neutralizing SARS-CoV-2 mAbs. (**a**) Schematic representation of engineered MSCs expressing neutralizing SARS-CoV-2 2–15, XGv347, and LY-CoV1404 mAbs. (**b**) Normalized binding to S1 and RBD of SARS-CoV-2 for mAbs secreted by MSCs clones (triangles: green, S1-binding; gray, not S1-binding; inverted triangles: green, RBD-binding; gray, not RBD-binding, the cutoff line for positive: OD ratio > 0.2). OD, optical density in ELISA; WT: untransfected MSCs; C1: vector control; C2: wt control. See also Figure S1. (**c**) Quantification of the released mAbs levels by antigen (S1)-specific ELISA using the supernatants collected after 96 h of culture from the top 18 neutralizing MSCs clones (mean ± SD).

The publicly available sequences of three SARS-CoV-2-neutralizing mAbs, named 2–15, LY-CoV1404, and XGv347, respectively, were used as the candidate sequences of mAbs [24–26]. Infection-elicited antibody LY-CoV1404 and vaccine-elicited antibody XGv347 were the two mAbs that have the optimal neutralization ability against Omicron variants. Therefore, LY-CoV1404 and XGv347 were used as the candidate sequences of mAbs. In addition, mAbs 2–15 was selected as a negative control for Omicron neutralization. To express the heavy and light chains of the neutralizing mAbs simultaneously in cells, the heavy and light chains were connected by internal ribosome entry site (IRES) and inserted into plasmids (pcDNA3.1) whose promoter region was modified for EF-1a (Fig. 1a). The sequence of signal peptide was added to the heavy and light chains of mAbs, respectively (Fig. 1a), for enhancing neutralizing antibody secretion. To verify the function of SARS-CoV-2 mAbs, 293T cells were transfected with corresponding recombinant plasmids. The binding capacity of secreted mAbs with spike 1 (S1) and receptor-binding domain (RBD) protein of SARS-CoV-2 were detected in supernatant post transfection. As expected, all three SARS-CoV-2 mAbs could potently and specifically bind to the RBD and S1 proteins (Fig. S1). Then the clinical-grade human umbilical cord derived MSCs transfected with empty plasmid was used for the generation of a vector control cell line. The untransfected MSCs were served as negative control cells. The recombinant plasmids were transfected into MSCs by electroporation and the positive stable cell clones were screened out by verifying the secretions of SARS-CoV-2-neutralizing mAbs in the culture supernatant. Herein, we obtained 45 MSC clones, of which all 12 clones transfected with the 2–15 mAb plasmid (2-15-MSCs), 10 out of 15 MSCs clones transfected with the LY-CoV1404 mAb plasimid (LY-CoV1404-MSCs), and 17 out of 18 MSC clones transfected with the XGv347 mAb plasmid (XGv347-MSCs) could successfully secret mAbs which recognize S1 and RBD (Fig. 1b). According to the concentration of mAbs secreted by MSC clones, we selected top 3 MSCs clones transfected with the 2–15 mAb plasmid, top 5 MSCs clones transfected with the XGv347 mAb plasmid, and top 10 MSCs clones transfected with the LY-CoV1404 mAb plasmid. Subsequently, the neutralizing mAb secretions of the top 18 MSCs clones were quantified. The mAb concentrations secreted from 2-15-MSCs clones in culture supernatant were between 103.1 and 128.5 ng/ml, meanwhile the mAbs concentration of LY-CoV1404-MSCs clones and XGv347-MSCs clones were ranged from 13 to 64 ng/ml and from 77 to 96 ng/ml, respectively (Fig. 1c).

### Omicron binding properties of SARS-CoV-2 mAbs secreted by engineered MSCs clones

**Fig. 2:**
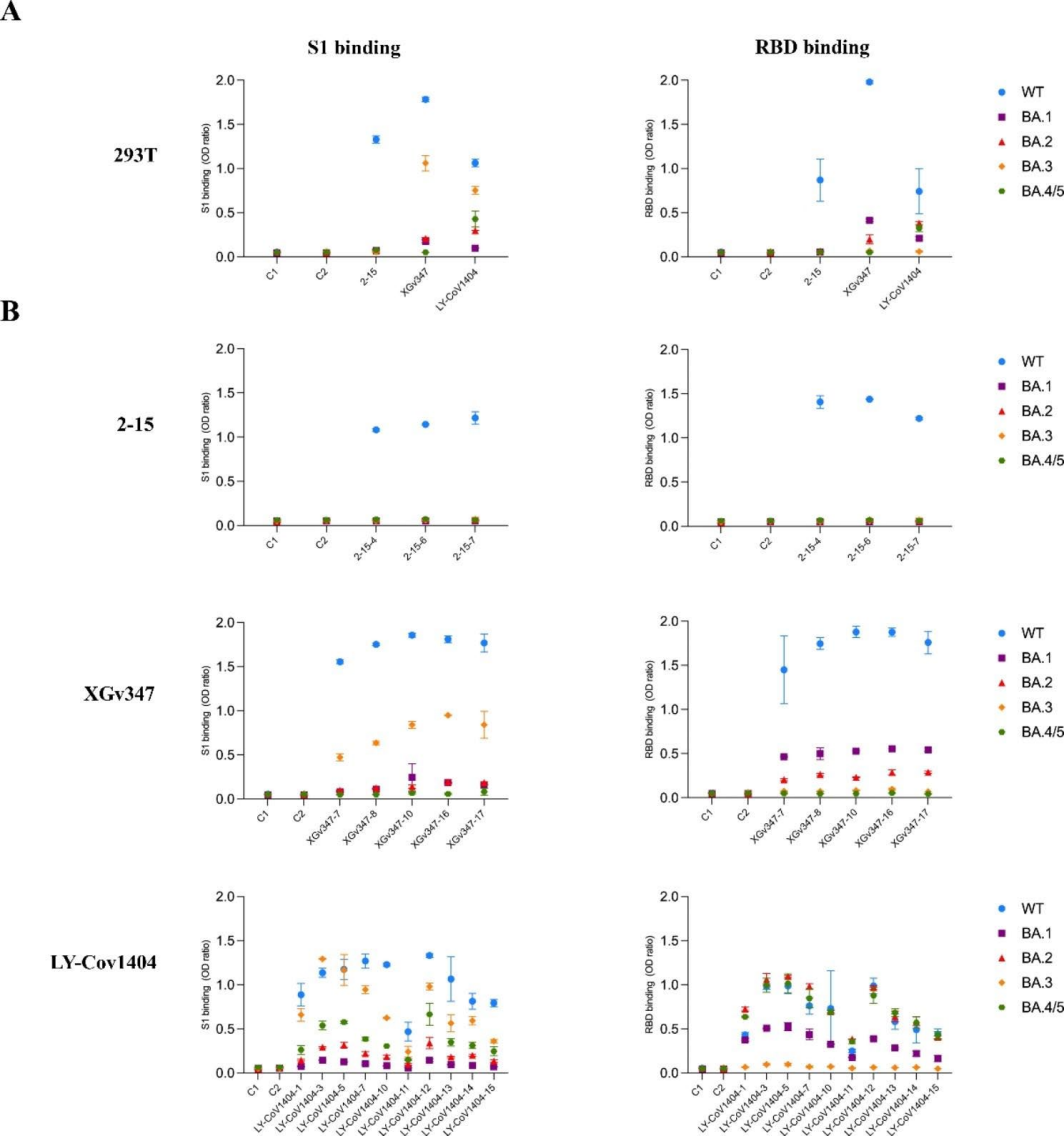
Binding affility of three kinds of neutralizing mAbs, 2–15, XGv347, and LY-CoV1404 to SARS-CoV-2 S1 and RBD mutants. (**a**) Three neutralizing mAbs secreted by 293T cells bound to SARS-CoV-2 S1 and RBD mutants including BA.1, BA.2, BA.3, and BA.4/5 by ELISA (mean ± SD). (**b**) The mAbs secreted by the top 18 neutralizing MSCs clones bound to SARS-CoV-2 S1 and RBD mutants including BA.1, BA.2, BA.3, and BA.4/5 in ELISA (mean ± SD).

To further characterize the functional activity of SARS-CoV-2 mAbs, we explored the ability of neutralizing mAbs secreted in culture supernatant by 18 MSCs clones to bind Omicron subvariant Spike and RBD proteins. All the secreted mAbs from LY-CoV1404-MSCs clones could bound to Omicron variants S1 and RBD except BA.3 RBD, whereas all secreted mAbs from 2-15-MSCs clones did not bind to Omicron subvariants S1 and RBD. The secreted mAbs from XGv347-MSCs clones bound well to BA.3 S1, BA.1 RBD and BA.2 RBD, weakly bound to BA.3 RBD, BA.1 S1 and BA.2 S1, and failed to recognize BA.4/5 S1 and RBD. In addition, all secreted mAbs from 18 neutralizing MSC clones have a similar binding capacity with the mAbs secreted by 293T for omicron subvariant S1 and RBD proteins (Fig. 2). Thus, these data suggested that our screened neutralizing MSC clones exhibited comparable binding strength to 293T-secreted mAbs. Subsequently, six MSC clones with most potent binding ability to S1 and RBD proteins were selected for pseudovirus neutralization.

### Neutralization potency of stable engineered MSCs clones against multiple omicron variants

**Fig. 3:**
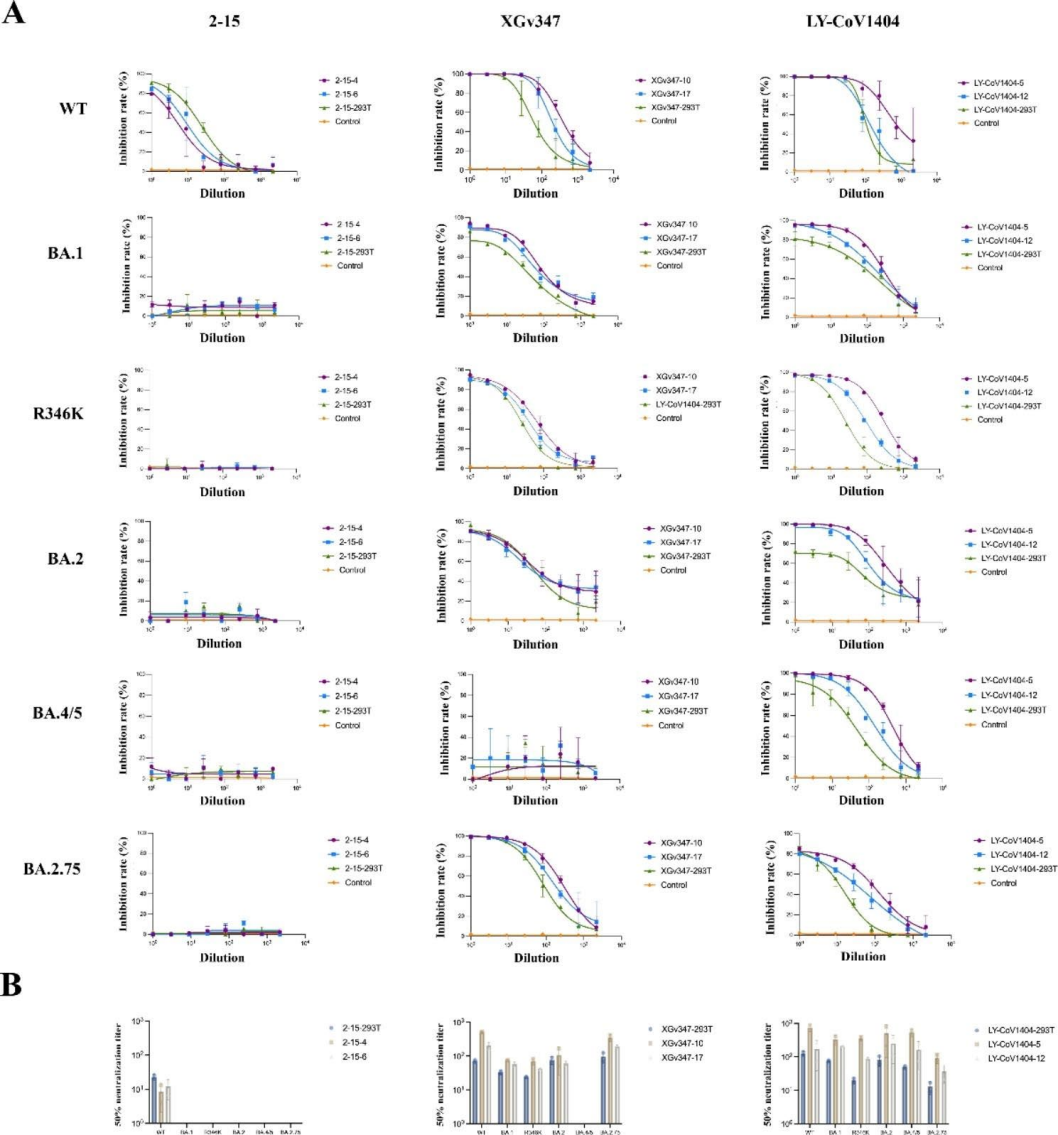
The neutralization titers of mAbs secreted by six most potent neutralizing MSCs clones against Omicron variants. (**a**) Neutralizing potency of three mAbs (2–15, XGv347, and LY-CoV1404) secreted by six most potent neutralizing MSC clones against pseudoviruses of wild type (WT) and the Omicron subvariants including BA.1, BA.1-R293K, BA.2, BA.4/5, and BA.2.75. The curve was presented by the inhibition percentage of SARS-CoV-2 pseudotyped viruses entry into host cells (mean ± SD). See also Figure S2. (**b**) The mean 50% neutralizing antibody titers derived from fitting the titration curves with a logistic regression model

To evaluate the neutralization potency of six engineered MSC clones with most potent binding ability to S1 and RBD proteins, pseudovirus neutralization assay was performed. We first determined the neutralization potency of six engineered MSCs clones using SARS-CoV-2 wild-type pseudotyped virus neutralization assay. The most potent MSCs clones of 2-15-6, XGv347-10 and LY-CoV1404-5, exhibited 50% neutralization titer for wild type pseudotyped virus assay was 12.12, 508.93, and 750.74, respectively (Fig. 3ab; S2a). To determine whether these engineered MSCs clones could secret mAbs to neutralize currently circulating Omicron subvariants, we then tested the neutralization potency of engineered MSC clones against five emerging Omicron variants including the BA.1, the BA.2, the BA.1-R346K, the BA.4/5, and the BA.2.75. Among all engineered MSCs clones tested, the clonotype XGv347 and LY-CoV1404 retained full neutralization potency against the Omicron subvariants and showed higher neutralization ability against the pseudotyped viruses than 293T-secreted mAbs (Fig. 3ab). Moreover, the clone XGv347-10 and LY-CoV1404-5 continued to display the highest potency against Omicron subvariants pseudovirus. Specifically, the neutralization potency of LY-CoV1404 against Omicron subvariants were higher than those of clonotype XGv347, in line with ELISA results of clonotype LY-CoV1404 binding with Omicron subvariants RBD. In addition, clonotype XGv347 which failed to recognize BA.4/5 S1 and RBD also did not show any neutralization activities for the BA.4/5 subvariant. Recently, a new Omicron variant BA2.75 has aggravated the pandemic situation. We then tested the neutralization ability of these MSCs clones against variant BA2.75. Notably, despite the clonotype XGv347 failed to neutralize the BA.4/5 subvariant, it presented higher neutralization ability against the BA.2.75 subvariant than clonotype LY-CoV1404, with 50% neutralization titer of 358.26, 193.32 in clonotype XGv347 and 50% neutralization titer of 94.63, 36.18 L in clonotype LY-CoV1404, respectively (Fig. S2a). Moreover, we hypothesized that the combination of natural infection-elicited antibodies and vaccine-elicited antibodies might synergistically improve the neutralizing capacity mediated by differences in the ACE2 binding site. Therefore, we tested the neutralization potency of combination of the strongest neutralizing clones (XGv347-10 and LY-CoV1404-5), but there was no improvement on neutralization potency (Fig. S2b). Together, our data showed that the screened engineered MSC clones retain their potency against Omicron subvariants, including currently circuiting variants including BA.4/5 and BA.2.75.

### Functional validation of engineered MSCs in vitro

**Fig. 4:**
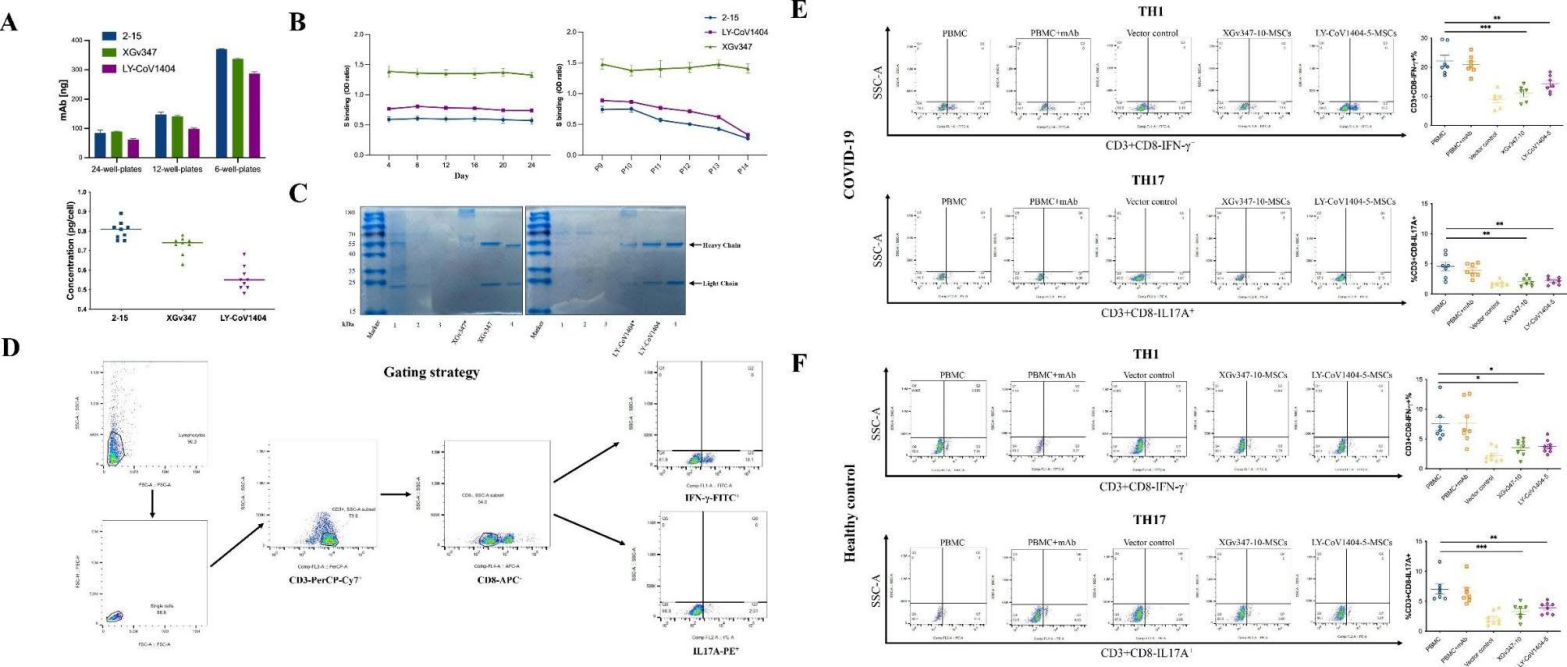
Functional validation of engineered MSCs in vitro. (**a**) The quantification of SARS-CoV-2 mAbs released by a single MSC clone (pg/cell) was performed by ELISA. MSCs were seeded at limiting cell densities and the level of antibody (ng) in culture supernatant (6-well-plates, 12-well-plates, and 24-well-plates) was determined after 96 h of culture (mean ± SD from three wells of one experiment). (**b**) The levels of SARS-CoV-2 mAbs released by screened positive MSCs clones at different time points (left; changing medium every 4 days without subculturing) and at different passages (right) (mean ± SD from three wells of one experiment). (**c**) Reducing SDS-PAGE analysis of SARS-CoV-2 mAbs released by MSCs (XGv347-10 and LY-CoV1404-5). Lane 1: Original cell culture supernatant, Lane 2: Supernatant after bind with protein G, Lane 3: Washing liquid, Lane 4: Purified IgG as positive control. *non-reducing SDS-PAGE. (**d**) Flow cytometry gating of Th1 and Th17 cells. Gates to exclude debris and cell aggregates in FSC-A/SSC-A and FSC-A/FSC-H plots. Representative flow cytometry gating strategy correspond to Th1(IFN-γ–FITC+) and Th17(IL-17 A–PE+). (**e**) Representative flow cytometry of Th1 and Th17 cells overlaid on total CD3 + T cells. Percentage of Th1 and Th17 cells quantified by expression of IFN-r and lL-17 A, respectively in acute COVID-19 (n = 8). **p < 0.01, ***p < 0.001. (**f**) Representative flow cytometry of Th1 and Th17 cells overlaid on total CD3 + T cells. Percentage of Th1 and Th17 cells quantified by expression of IFN-r and lL-17 A, respectively in unexposed (n = 8). **p < 0.01, ***p < 0.001

To evaluate the ability of mAbs secretion by the top 2 MSCs clones (XGv347-10 and LY-CoV1404-5), we first determined the level of mAbs secreted by a single modified MSC clone. The mAbs secretion potency of MSCs clones remained stable in different well-plates, and the single MSC of either XGv347-10 or LY-CoV1404-5 released 0.728 pg and 0.557 pg mAbs within 96 h, respectively (Fig. 4a). We then tested the impact of time and passage on mAbs secretion. To investigate the mAbs secretion stability of MSCs clones, after seeding cells at passage 4, we changed medium every 4 days for 24 days and assayed the mAbs concentrations in medium although the cell confluence reached 100% and subculturing was not performed. The results showed the concentrations of three secreted mAbs could keep stable without subculturing during 24 days. While the concentration of mAbs secretion was declined with the increase of passages, except for the clone XGv347-10 (Fig. 4b). Meanwhile, the integrity of mAbs secreted by the clone XGv347-10 and LY-CoV1404-5 was confirmed by reducing and non-reducing sodium dodecyl-sulfate polyacrylamide gel electrophoresis (SDS-PAGE) (Fig. 4c).

In light of the ability of MSCs to suppress the differentiation of CD4 + T cells into Th1 and Th17 subpopulations [27], we examined whether the engineered MSCs still maintained such immunomodulatory effects in COVID-19. Here we assessed the immunomodulatory effects of engineered MSCs (XGv347-10 and LY-CoV1404-5) by co-culturing with PBMCs from healthy and SARS-CoV-2-infected individuals. The characteristics of COVID-19 patients were shown in Tab. S1. As expected, the two MSCs clone significantly inhibited CD4 + T cells from PBMC of COVID-19 patients to differentiate into Th1 and Th17 subpopulation and promoted the maturation of Tregs subpopulation in PBMCs induced by IL-2 (Fig. 4e, S3), whereas there is no immunomodulatory effects for SARS-CoV-2 mAbs treatment alone. Similar results were observed in non-COVID-19 (Fig. 4f). Notably, significant Th1 activation were observed in acute COVID-19 patients when compared with healthy control (HC) (22.64% vs. 7.82%, p < 0.0001), consistent with previous reports [28]. Our data indicated that the engineered MSCs were able to well-coordinate the hyperactivated T-cells responses caused by COVID-19.

### The pharmacokinetics and biodistribution of mAbs secreted by transplanted engineered MSCs in vivo

**Fig. 5:**
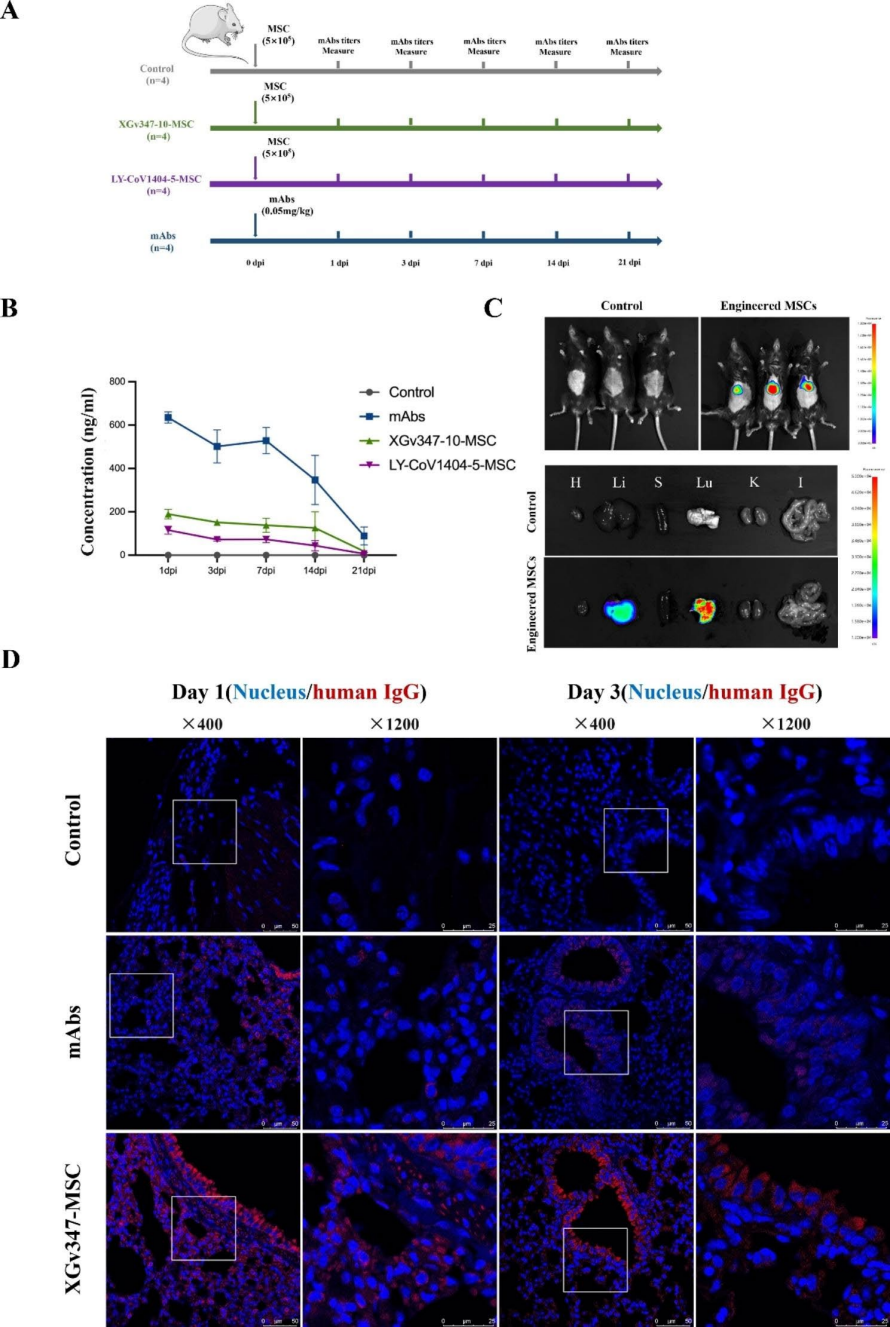
The in vivo expressions of SARS-CoV-2 mAbs with engineered MSCs delivery. (**a**) Schematic overview of the animal experiment. (**b**) Concentrations of mAbs in serum, n = 4. (**c**) Bio-imaging of mice body at day1 and mice were sacrificed at day1 post engineered MSCs administration and dissected organs were imaged (n = 3). Lu, lung; K, kidney; H, heart; S, spleen; Li, liver; I, intestines. (**d**) The lung tissues collected at 1 day and 3 days after the administration of engineered MSCs and SARS-CoV-2 mAbs were subjected to IFAs analysis for the expressions of SARS-CoV-2 mAbs using PE conjugated goat anti-human IgG (H + L). Bar scle = 50 μm or 25 μm. (n = 4 mice per group)

To characterize mAbs pharmacokinetics of transplanted engineered MSCs in vivo, the six to eight weeks-old BALB/c mice were administered with 5 × 10^5^ IU mAb-MSCs or commercial SARS-CoV-2 mAbs at 0.05 mg/kg (Fig. 5a). Antibody levels in serum were tested using ELISA at 1, 3, 7, 14, and 21 days post administration. As shown in Fig. 5b, the serological SARS-CoV-2 mAb level was markedly higher in mAbs group than that in engineered MSC groups at indicated time points, but declined rapidly, whereas the serum mAbs levels in engineered MSCs groups remained relatively more stable than that mAbs groups during the study period. Our results demonstrated that transplanted engineered MSCs could efficiently and constantly secret neutralizing antibody into serum in vivo.

To further assess the SARS-CoV-2 mAbs biodistribution in the mice transduced with engineered MSCs, we first assessed tissue distribution of engineered MSCs at day 1 post administration by conjugating the MSCs with DiR to enable in vivo imaging. As shown in Fig. 5c, the MSCs were concentrated mainly in lung and very few in other organs. Therefore, we further detected the SARS-CoV-2 mAb concentrations in lung. Immunofluorescence staining of lung tissues at different time points indicated that a substantially stronger staining of SARS-CoV-2 mAbs was detected after transplanting engineered MSCs, compared with alone administration of mAbs. (Fig. 5d, S4).

## Discussion

The continuous emergence of new SARS-CoV-2 subvariants highlights the urgent need to develop effective treatment for severe COVID-19, especially the elderly. MSCs possess several unique features, such as migration to inflammatory tissues and regulation of inflammatory environment, which make them highly attractive for an application as cellular weapons in COVID-19. In our study, modified recombinant plasmids were transfected into MSCs to secrete SARS-CoV-2-neutralizing mAbs. We have identified two mAb-MSC clones secreting effective neutralizing monoclonal antibodies that recognized not only WT RBD and S1 protein, but also some recently emerging Omicron variant RBD and S1 proteins including RBD and S1 BA.1, RBD and S1 BA.2, RBD and S1 BA.3, RBD and S1 BA.4/5. Consistent with their capabilities of binding S1 and RBD, two mAb-MSCs clones were able to neutralize SARS-CoV-2 wildtype and Omicron subvariants using pseudovirus neutralization assay. Moreover, we confirmed that a substantially greater amount of SARS-CoV-2-neutralizing mAbs could be delivered to lung via engineered MSCs than direct administration of commercial SARS-CoV-2 mAbs.

Understanding the levels of SARS-CoV-2-neutralizing mAbs secretion in engineered MSCs is of utmost importance. A recent study reported that the highest amount of mAbs released by a single MSC was 8.8 pg/cell [23]. In our study, a single modified MSCs (XGv347-10) released 0.728 pg SARS-CoV-2-neutralizing mAbs. Notably, the mAbs concentration in supernatants of MSCs clones 2–15 and XGv347 was higher than that of in transfected 293T cells (Fig. 1c). Moreover, gene-modified MSCs secreted the neutralizing mAbs in stable concentrations even after prolonged in vitro culture (Fig. 4c). In general, our study demonstrated the feasibility of employing MSCs as SARS-CoV-2-neutralizing mAbs production machines.

Since the recent emergence of B.1.1.529 (Omicron) variant was identified in South Africa in late 2021, it has evolved into four sub-lineages-BA.1, BA.2, BA.3, and BA.4/5 with BA.4/5 becoming dominant worldwide and raised concerns on immune escape from antibody recognition because of a larger number of mutations [29, 30]. A recent study has demonstrated that most clinically approved mAbs lose neutralizing activity against Omicron variant, and only LY-CoV1404 (bebtelovimab) demonstrated high neutralizing potency against all Omicron sub-lineages with half-maximal inhibitory concentration (IC_50_) ≤ 30 ng /ml [31]. In our study, the LY-CoV1404 secreted by modified MSCs showed high potency against all tested Omicron subvariants and still maintained neutralizing activity against recently emerged Omicron subvariant BA.2.75, which displayed a growth advantage over BA.4/5 [32]. Notably, the 50% neutralization titer of the clone LY-CoV1404-5 ranged from 94.63 to 750.74 and the mAbs concentration of the clone LY-CoV1404-5 was 59 ng/mL. Thus, the IC_50_ of the clone LY-CoV1404-5 ranging from 0.08 to 0.62 ng/mL was consistent with previous reports^31^(Tab. S2). Different most SARS-CoV-2-neutralizing mAbs isolated from convalescent COVID-19 patients, XGv347 was discovered from memory B cells in triple vaccination recipients, which showed high neutralizing activities against Omicron with an IC_50_ of 6 ng/mL [26]. The data was also consistent with our pseudovirus neutralization assay, in which the clone XGv347-10 had an IC_50_ value of 1.28 ng/mL. It was noteworthy that the clone XGv347-10 maintained neutralizing activity against BA.2.75 and had a higher neutralizing potency than the clone LY-CoV1404-5 with an IC_50_ value of 0.0027 ng/ml, while the clone LY-CoV1404-5 only had an IC_50_ value of 0.63 ng/ml. Hence, our results indicated that the mAbs secreted from MSCs presented comparable level of neutralizing potency compared to clinically approved antibodies, further supporting the utility of MSCs as antibody delivery vehicles against COVID-19.

While most Omicron infections are not severe, a substantial number of older individuals (> 65 years old) become life-threateningly ill, and require hospitalization, with increased rates of fatalities [33, 34]. As the understanding of immunity to COVID-19 is growing, the immunopathogenesis has become a major pathogenic factor of severe COVID-19 [35, 36]. The coordinated T-cells and antibody responses are protective. T-cells in mild COVID-19 are increased and cross-talking well, while in critical COVID-19, the peripheral CD4 and CD8 T cells are less abundant and dysregulated, their status was hyperactivated, manifested by increase of Th17 and Th1 cells [37, 38], thereby exacerbating inflammation and tissue damage. In our study, we collected PBMC samples from 8 older individuals (> 65 years old) with acute COVID-19 to assay their T cell populations by flow cytometric analysis. We found that the counts of peripheral Th1 cells were substantially increased in acute COVID-19 patients, as evidenced by the proportions of IFN-γ (in acute COVID-19 VS in healthy control), which confirmed the overactivation of T cells in acute COVID-19. Moreover, the MSCs clones XGv347-10 and LY-CoV1404-5 significantly inhibited Th1 subpopulation differentiation of PBMCs in acute COVID-19 in vitro. Additionally, after co-culturing with engineered MSCs, there was a decreased concentration of highly proinflammatory Th17 in CD4 T cells. Our results implied that engineered MSCs could coordinate immune hyperresponsiveness caused by COVID-19 in older individuals.

The morbidity and mortality in COVID-19 were largely resulted from alveolar injury manifested as acute respiratory distress syndrome (ARDS) in severe disease [39]. At present, there was no effective method to improve the concentration of SARS-CoV-2-neutralizing mAbs in the lung, leading to limited effectiveness of neutralizing mAbs in severe SARS-CoV-2 patients [40]. Previously we and others reported that MSCs first resided in the lung after infusion and then migrate to the parenchyma and airways [41–44]. Therefore, we hypothesized that the SARS-CoV-2-neutralizing mAbs secreted by MSCs might enrich in lung. As expected, our study demonstrated that SARS-CoV-2-neutralizing mAbs can be efficiently delivered to lung via pulmonary residence of engineered MSCs.

This study also has some limitations. Firstly, the safety of mAbs producing MSC-related therapies remains elusive for clinical applications. The principal risks of gene-modified MSCs are tumorigenicity. MSCs have the potential ability to develop into tumors, and some studies have shown that Ewing’s sarcoma cells are derived from MSCs [45]. Fortunately, tumorigenicity were rare according to clinical trials based on MSC therapy [46]. But we still should carry out comprehensive risk assay to ensure the safety of mAbs producing MSCs in clinic use. Secondly, low levels of mAbs produced by MSCs may rapidly select escape mutants due to insufficient neutralization. Therefore, we will further modify the MSCs-mAbs platform to increase the mAbs secretion of MSCs. Finally, the current study lacks in vivo experiments to demonstrate its efficacy. We will further use mouse models with SARS-CoV-2 infection to verify the efficacy of this MSCs-mAbs platform in a legally qualified infectious disease laboratory.

In summary, our study presents a new strategy to effectively treat older and immunocompromised SARS-CoV-2 infected patients and also provides alternative effective approach for prevention against other respiratory pathogens with the utility of engineered MSCs as the delivery platform.

## Materials and methods

### Cell culture

Clinical-grade human umbilical cord derived MSCs were used in this study that were greatly optimized in our previous research [28] and the entire culture of clinical‑grade MSCs conformed to GMP quality standards. HEK293T were cultured in Dulbecco’s modified Eagle’s medium (DMEM) (10% FBS, 100 µg/ml penicillin/streptomycin) in 5% CO2 at 37 °C. All procedures involving human subjects in this study were approved by the Research Ethics Board of Nanjing Drum Tower Hospital (Approval number: GCP-SCP/17/2).

### Plasmid construction

Infection-elicited antibody LY-CoV1404 and vaccine-elicited antibody XGv347 were the two mAbs that have the best neutralization ability against Omicron variants. Therefore, LY-CoV1404 and XGv347 were used as the candidate sequences of mAbs. In addition, we chose mAbs 2–15 as a negative control for neutralizing Omicron. The coding sequences of the heavy and light chains of 2–15, XGv347, and LY-CoV1404 mAb (GenBank: MT712281.1 and MT712298.1, PDB: 7WEC-I and 7WEC-L, PDB: 7MMO-D and 7MMO-E, respectively) were in vitro synthesized from GenScript, China, and inserted into plasmids (pcDNA3.1). To express the heavy and light chains of mAb simultaneously, a polycistronic mRNA (IRES) was inserted between the heavy and light chains of mAb into the plasmids. All the plasmids were confirmed by sequencing analysis before the subsequent experiments.

### Generation of recombinant human monoclonal antibodies

For mAbs expression, recombinant plasmids were transiently transfected into human embryonic kidney cells (HEK293T). Two days later, cell culture supernatants were harvested, and were purified using Protein G Sepharose beads (Thermo Scientific), dialyzed against PBS and sterile-filtered using 0.22 mm filter units (GE Healthcare). Ig concentrations were determined and used for in vivo experiments. Ig integrity was examined by sodium dodecylsulfate polyacrylamide gel electrophoresis (SDS-PAGE).

### Engineered MSCs generation and screening

To generate the mAb-MSCs, 1 × 10^6^ clinical-grade MSCs at passage 2 (P2) in 100ul MSCs nucleofector solution (cat. no. PT-2501, Lonza, Germany) were electroporated with 2 µg of each plasmid, in a cuvette with the Nucleofector apparatus (Lonza) at U-23 program (for high transfection efficiency). The electroporated cells were removed from the cuvette immediately and seeded in a 10-cm dish, and then incubated at 37 °C in 5% CO_2_. 1.2 µg/ml geneticin was used for stable clone screening. After 2 weeks, cell clones were observed under stereomicroscope for expansion culture, and each MSCs clone represented a cell line. When each MSCs clone grew to 90% confluence, the supernatant was collected and mAbs secretion level was determined by ELISA.

### Enzyme-linked immunosorbent assay (ELISA)

WT and Omicron variants (BA.1, BA.2, BA.3, BA.4/5) S1 and RBD protein at 50 ng per well was coated in 96-well microtiter plates (Corning, Cat no: 9018) overnight at 4 °C. The antigen coated plates were then blocked with 200 µl blocking buffer (5% bovine serum albumin in PBS) at 37 °C for 1 h and washed by three times of PBST (PBS with 0.05% Tween 20). Then cell culture supernatants were added and incubated at 37 °C for 1.5 h. The plate was washed with PBST for five times and incubated with 0.08 mg/mL goat anti-human IgG (H + L)/HRP (JACKSON) for 1 h incubation at 37 °C. Plates were washed five times with PBST. Finally, the TMB substrate (Abcam) was added and incubated for 3 min before the reaction was stopped using 1 M sulfuric acid. Optical density (OD) values were measured at 450 nm.

### Reduced and non-reduced PAGE

For denaturing SDS-PAGE and non-reduced PAGE, protein samples were mixed with loading buffer and no denaturing protein loading buffer, and then heated at 100 °C for 10 min. These samples were then loaded into 12% gels with pre-stained SDS-PAGE Standards. Electrophoresis was performed for approximately 120 min using a constant voltage (120 V) in running buffer. The gels were stained using Coomassie Brilliant Blue (Yeasen) and imaged.

### Pseudovirus neutralization assay

Neutralization assays were performed by incubating pseudoviruses with cell culture supernatants at 37 °C for one hour. The 96-well plates were coated with Huh7 cells at density of 10,000 per well. The mixture was added to cultured cells and after 24 h of incubation in a 5% CO2 environment at 37 °C, washed with PBS for two times, the luminescence was measured using luciferase substrate (One-GloTM Luciferase assay system, Promega, E6120). Percent of inhibition was calculated as relative reduction of relative light unit (RLU) compared with the virus control wells. Data were non-linear fitted and ID50 was calculated using four parameters regression in GraphPad Prism.

### Flow cytometry

All human blood samples were collected according to the doctor’s instruction from Nanjing Drum Tower Hospital, China. Peripheral blood mononuclear cells (PBMCs) were isolated immediately from fresh blood.

MSCs were co-cultured with PBMCs (MSCs/PBMCs ratio, 1:10) in 6-well plates. After co-culturing for 3 days, PBMCs were collected and incubated with 1× compound stimulant of cocktail (Invitrogen, USA) for 5 h at 37 °C according to the instruction of Kit. Then, for the surface stain, PBMCs were resuspended in PBS and incubated with CD3-Percp and CD8-APC (BD, USA) at 37 °C in the dark for 15 min. Following surface staining, cells were washed with PBS and resuspended in Cell Fixation/Permeabilization kit (FMS, China) incubated at room temperature for 30 min in the dark. Cells were then washed with PBS and stained with intracellular antibodies (IFN-r -FITC and IL-17 A-PE) for 20 min in the dark. After staining, cells were washed once with PBS and resuspended in 100ul PBS. Data were acquired on a flow cytometry (BD FACSAriaTM, USA), and analyzed with Flowjo.

### Pharmacokinetic analysis

For XGv347, 0.05 mg/kg XGv347 were administrated into mice from tail. The mAb-MSCs were administrated by tail injection at a dose of 5 × 10^5^. After administration, four mice were sacrificed to collect plasma at the following timepoints: 1dpi, 3dpi, 7dpi, 14dpi, 21dpi (Fig. 5a). The blood was centrifuged at 3,500 x g for 10 min and then transferred into 1.5 mL tubes and stored at -80 °C. The lung was weighed, homogenized, and then centrifuged at 12,000 rpm. The supernatant was harvested and stored at -80 °C for further quantified. Antibody levels in each sample were quantified by ELISA. In brief, WT RBD protein at 50 ng per well was coated in 96-well microtiter plates (Corning, Cat no: 9018) overnight at 4 °C. The antigen coated plates were then blocked with 200 µl blocking buffer (5% bovine serum albumin in PBS) at 37 °C for 1 h and washed by three times of PBST (PBS with 0.05% Tween 20). 50 ul of mice plasma in PBS at a dilution of 1:10 was added and incubated at 37 °C for 1.5 h. The plate was washed with PBST for five times and incubated with 0.08 mg/mL goat anti-human IgG (H + L)/HRP (JACKSON) for 1 h incubation at 37 °C. The plate was washed with PBST for five times and the OD) values were measured by recording the absorbance at 450 nm after incubation with TMB substrate (Abcam) for 3 min. Gradient serially diluted purified antibodies were used to generate quantitative standard curve and fitted by a four-parameter logistic model. The antibody concentration in lung and plasma was calculated from the standard curve.

### Near-infrared fluorescence imaging

Imaging MSC in PBS were incubated with 1 mM DiR (Invitrogen, USA), which is a fluorescent lipophilictracer, at room temperature for 30 min. Then, DiR-labeled MSCs were administered to the mice through the tail vein. The mice were anesthetized and maintained in a prone position. The fluorescence signals in the tissues were quantified at the 24 h time point (n = 3 for each group). using IVIS Lumina III system (PerkinElmer, UK). For ex vivo imaging, mice were sacrificed at the 24 h time point (n = 3 for each group). Organs including heart, lungs, liver, kidneys, spleen, and intestine were collected and weighed. The collected organs were placed on black plastic spacers and imaged using the IVIS Lumina III system (PerkinElmer, UK).

### Biodistribution analysis

Organ samples from mice at 1 and 3 dpi were fixed with 4% paraformaldehyde, embedded in paraffin followed by sagittal sections of 4-µm thickness on a microtome, and mounted on slides. For detection of SARS-CoV-2 mAbs secreted by engineered MSCs in fixed organs, immunofluorescence assay (IFA) was conducted. Briefly, the slides were deparaffinized, rehydrated and experienced heat induced antigen retrieval with EDTA (pH 8.0) in a microwave oven. Then tissues were uniformly covered with 5% BSA for incubation at room temperature for 1 h followed by further incubation with anti-human IgG H&L (PE) at 1:100 dilution. After washing in PBS, slides were stained with DAPI (Beyotime) at 1:100 dilution. The image information was collected using a Pannoramic MIDI system (3DHISTECH, Budapest) and FV1200 confocal microscopy (Olympus).

### Electronic Supplementary Material

Below is the link to the electronic supplementary material.

**Additional File: Figure S1** Normalized binding to S1 and RBD of SARS-CoV-2 for mAbs secreted by 293T cells. OD, optical density in ELISA. Related to Fig. 1

**Additional File: Figure S2** Neutralizing abilities of the mAbs secreted by most potent MSCs clones. (a) Mean 50% neutralizing antibody titers of the neutralizing mAbs, Related to Fig. 3. (b) Neutralizing potency of combination of neutralizing clones (LY-CoV1404-5: XGv347-5) against pseudoviruses of wild type (WT) and the variant BA.1. The curve is presented by the inhibition percentage of SARS-CoV-2 pseudotyped viruses entry into host cells (mean ± SD from two independent measurements). Related to Fig. 3

**Additional File: Figure S3** Representative flow cytometry of Treg cells overlaid on total CD4^+^ T cells. (a) Flow cytometry gating of Treg and dying cells. Gates to exclude debris and cell aggregates in FSC-A/SSC-A and FSC-A/FSC-H plots. Representative flow cytometry gating strategy correspond to Treg (FoxP3-PE^+^) and dying cells (FVS–APC+). Related to Fig. 3. (b,c) Percentage of Treg cells quantified by expression of FoxP3, in acute COVID-19 (n = 8). **p. <. 0.01, *p < 0.05. Related to Fig. 3

**Additional File: Figure S4** The lung tissues collected at 21 days after the administration of engineered MSCs and SARS-CoV-2 mAbs were subjected to IFA analysis to stain for the expression of SARS-CoV-2 mAbs using PE conjugated goat anti-human IgG (H + L). Scale bars represent 75 µm. (n = 4 mice per group). Related to Fig. 5

**Additional File: Table S1** The characteristics of COVID-19 patients. Related to Fig. 4

**Additional File: Table S2** The neutralization potency (IC_50_) of SARS-CoV-2 mAbs (2–15, XGv347 and LY-CoV1404)

## Data Availability

All data generated or analyzed during this study are included in this published article. The datasets used and/or analyzed during the current study are available from the corresponding author on reasonable request.

## Abbreviations

SARS-CoV-2: Severe acute respiratory syndrome coronavirus 2
mAbs: Monoclonal antibodies
MSC: Mesenchymal stromal cell
ARDS: Acute respiratory distress syndrome
IRES: Internal ribosome entry site
S1: Spike 1
RBD: Receptor-binding domain
ELISA: enzyme-linked immunosorbent assay
