# Supplementary material for: Engineered clinical-grade mesenchymal stromal cells combating SARS-CoV-2 omicron variants by secreting effective neutralizing antibodies
Source: Cell Biosci. 2023 Aug 31;13:160. doi: 10.1186/s13578-023-01099-z (PMC10470189; doi:10.1186/s13578-023-01099-z)
Supplement: Supplementary file 1 — Additional File: Figure S1 Normalized binding to S1 and RBD of SARS-CoV-2 for mAbs secreted by 293T cells. OD, optical density in ELISA. Related to Fig. 1 [file 13578_2023_1099_MOESM1_ESM.docx]

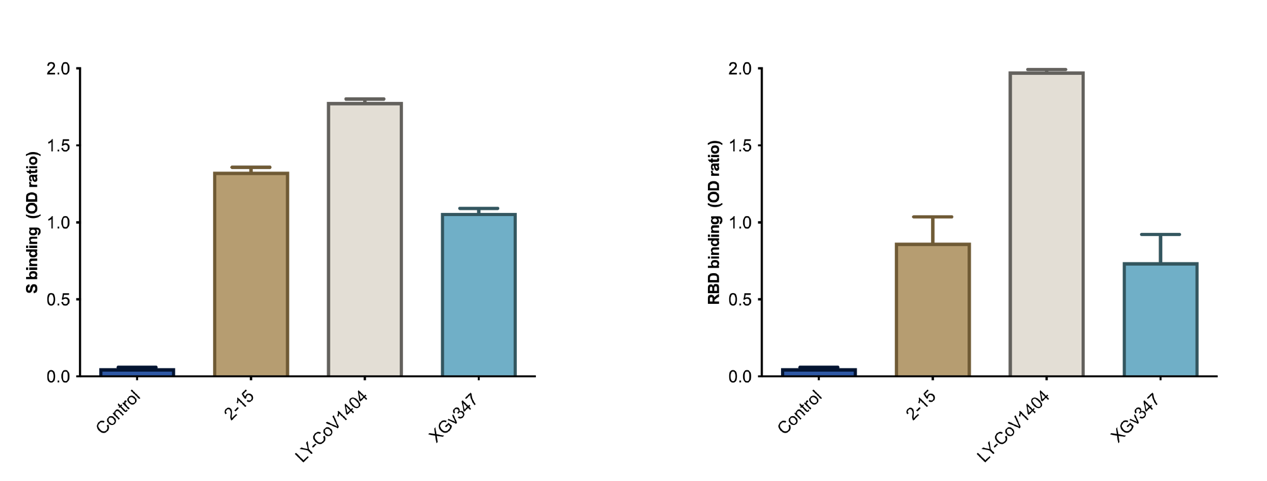


**Figure S1** Normalized binding to S1 and RBD of SARS-CoV-2 for mAbs secreted by 293T cells. OD, optical density in ELISA. Related to Figure 1
