# Supplementary material for: Engineered clinical-grade mesenchymal stromal cells combating SARS-CoV-2 omicron variants by secreting effective neutralizing antibodies
Source: Cell Biosci. 2023 Aug 31;13:160. doi: 10.1186/s13578-023-01099-z (PMC10470189; doi:10.1186/s13578-023-01099-z)
Supplement: Supplementary file 2 — Additional File: Figure S2 Neutralizing abilities of the mAbs secreted by most potent MSCs clones. (a) Mean 50% neutralizing antibody titers of the neutralizing mAbs, Related to Fig. 3. (b) Neutralizing potency of combination of neutralizing clones (LY-CoV1404-5: XGv347-5) against pseudoviruses of wild type (WT) and the variant BA.1. The curve is presented by the inhibition percentage of SARS-CoV-2 pseudotyped viruses entry into host cells (mean ± SD from two independent measurements). Related to Fig. 3 [file 13578_2023_1099_MOESM2_ESM.docx]

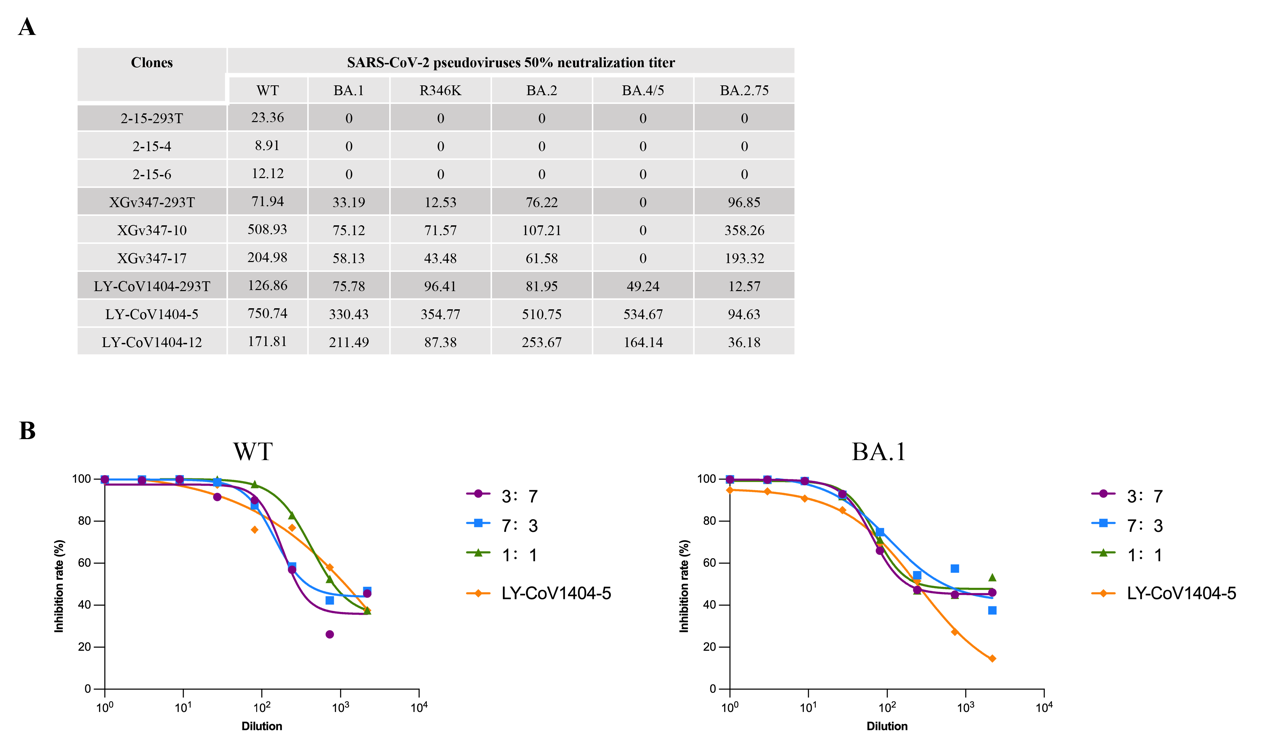


**Figure S2** Neutralizing abilities of the mAbs secreted by most potent MSCs clones

1. Mean 50% neutralizing antibody titers of the neutralizing mAbs, Related to Figure 3
2. Neutralizing potency of combination of neutralizing clones (LY-CoV1404-5: XGv347-5) against pseudoviruses of wild type (WT) and the variant BA.1. The curve is presented by the inhibition percentage of SARS-CoV-2 pseudotyped viruses entry into host cells (mean ± SD from two independent measurements). Related to Figure 3
