# Supplementary material for: Engineered clinical-grade mesenchymal stromal cells combating SARS-CoV-2 omicron variants by secreting effective neutralizing antibodies
Source: Cell Biosci. 2023 Aug 31;13:160. doi: 10.1186/s13578-023-01099-z (PMC10470189; doi:10.1186/s13578-023-01099-z)
Supplement: Supplementary file 3 — Additional File: Figure S3 Representative flow cytometry of Treg cells overlaid on total CD4+ T cells. (a) Flow cytometry gating of Treg and dying cells. Gates to exclude debris and cell aggregates in FSC-A/SSC-A and FSC-A/FSC-H plots. Representative flow cytometry gating strategy correspond to Treg (FoxP3-PE+) and dying cells (FVS–APC+). Related to Fig. 3. (b,c) Percentage of Treg cells quantified by expression of FoxP3, in acute COVID-19 (n = 8). **p. <. 0.01, *p < 0.05. Related to Fig. 3 [file 13578_2023_1099_MOESM3_ESM.docx]

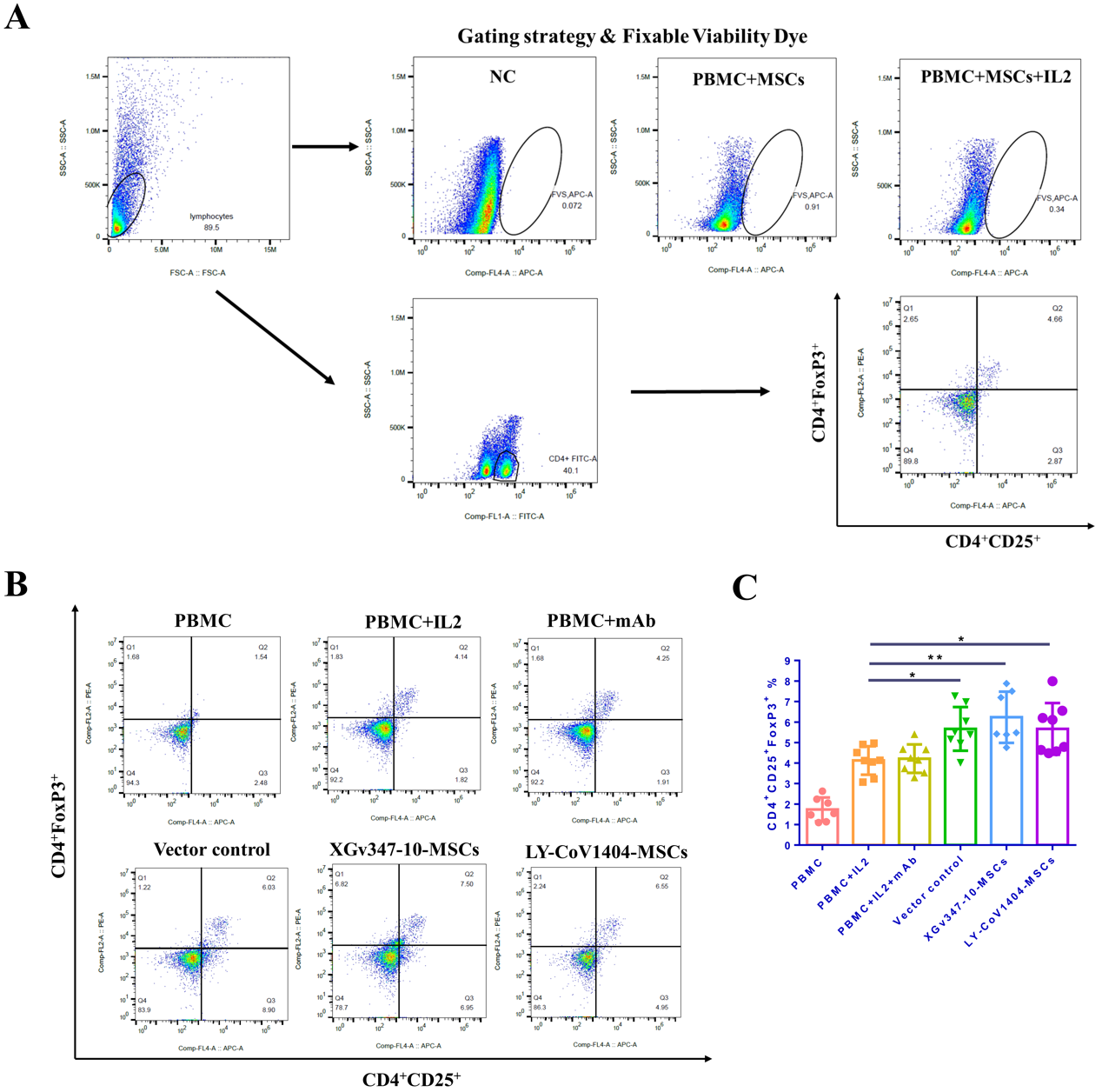


**Figure S3** Representative flow cytometry of Treg cells overlaid on total CD4^+^ T cells

1. Flow cytometry gating of Treg and dying cells. Gates to exclude debris and cell aggregates in FSC-A/SSC-A and FSC-A/FSC-H plots. Representative flow cytometry gating strategy correspond to Treg (FoxP3-PE^+^) and dying cells (FVS–APC+). Related to Figure 3

(b,c) Percentage of Treg cells quantified by expression of FoxP3, in acute COVID-19 (n = 8). **p. <. 0.01, *p < 0.05. Related to Figure 3
