# Supplementary material for: Engineered clinical-grade mesenchymal stromal cells combating SARS-CoV-2 omicron variants by secreting effective neutralizing antibodies
Source: Cell Biosci. 2023 Aug 31;13:160. doi: 10.1186/s13578-023-01099-z (PMC10470189; doi:10.1186/s13578-023-01099-z)
Supplement: Supplementary file 4 — Additional File: Figure S4 The lung tissues collected at 21 days after the administration of engineered MSCs and SARS-CoV-2 mAbs were subjected to IFA analysis to stain for the expression of SARS-CoV-2 mAbs using PE conjugated goat anti-human IgG (H + L). Scale bars represent 75 µm. (n = 4 mice per group). Related to Fig. 5 [file 13578_2023_1099_MOESM4_ESM.docx]

**
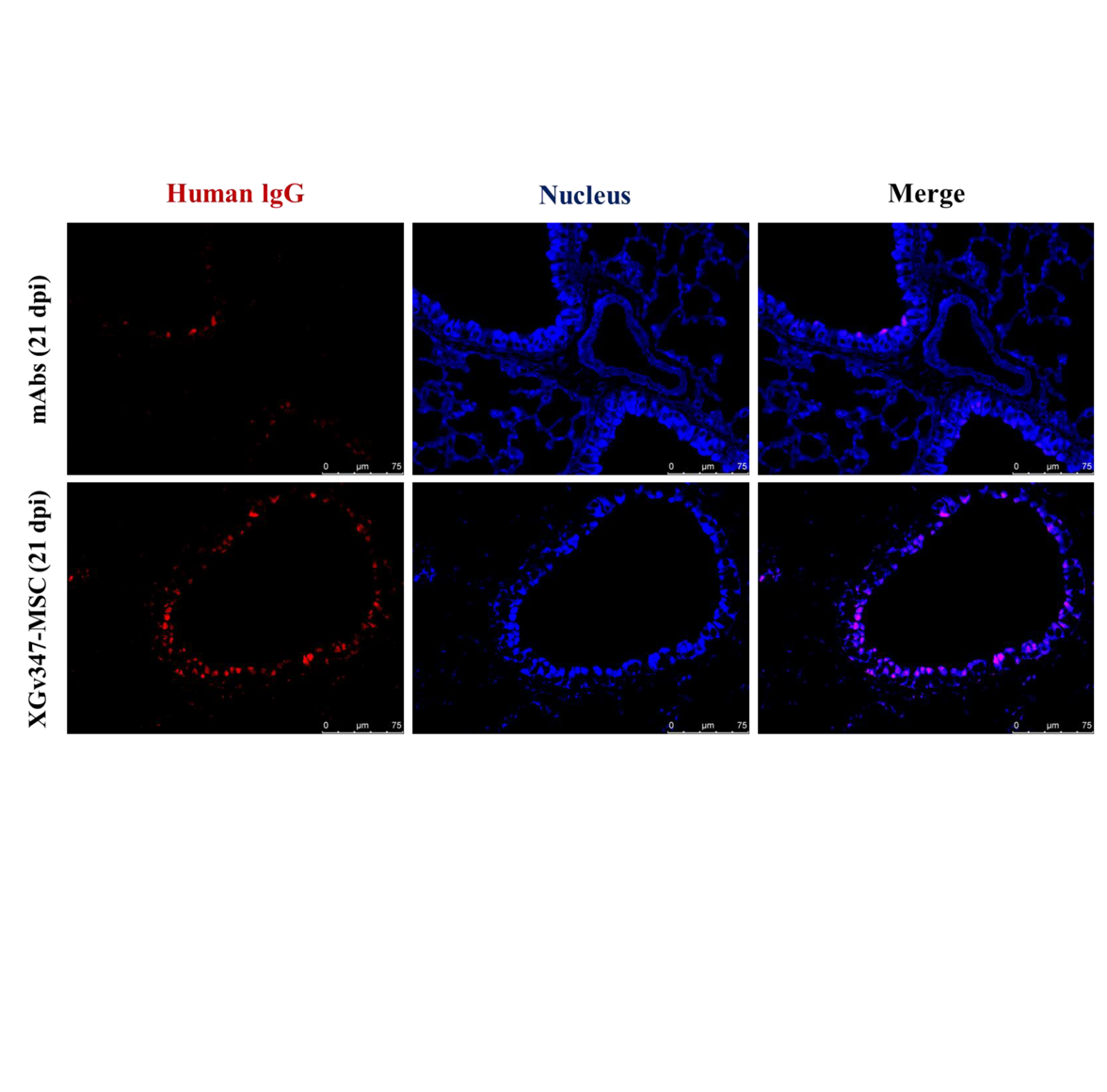
**

**Figure S4** The lung tissues collected at 21 days after the administration of engineered MSCs and SARS-CoV-2 mAbs were subjected to IFA analysis to stain for the expression of SARS-CoV-2 mAbs using PE conjugated goat anti-human IgG (H + L). Scale bars represent 75 μm. (n= 4 mice per group). Related to Figure 5
