# Supplementary material for: Engineered clinical-grade mesenchymal stromal cells combating SARS-CoV-2 omicron variants by secreting effective neutralizing antibodies
Source: Cell Biosci. 2023 Aug 31;13:160. doi: 10.1186/s13578-023-01099-z (PMC10470189; doi:10.1186/s13578-023-01099-z)
Supplement: Supplementary file 5 — Additional File: Table S1 The characteristics of COVID-19 patients. Related to Fig. 4 [file 13578_2023_1099_MOESM5_ESM.docx]

| Patients | Age | Sex | Comorbidity | Corticosteroids | Disease severity |
| --- | --- | --- | --- | --- | --- |
| 1 | 80 | Male | Yes | No | Severe |
| 2 | 90 | Male | Yes | No | Severe |
| 3 | 67 | Female | No | Yes | Severe |
| 4 | 95 | Female | Yes | No | Severe |
| 5 | 78 | Male | No | Yes | Severe |
| 6 | 85 | Male | Yes | No | Severe |
| 7 | 89 | Male | Yes | No | Severe |
| 8 | 92 | Male | Yes | No | Severe |

**Table S1** The characteristics of COVID-19 patients. Related to Figure 4
