# Supplementary material for: Engineered clinical-grade mesenchymal stromal cells combating SARS-CoV-2 omicron variants by secreting effective neutralizing antibodies
Source: Cell Biosci. 2023 Aug 31;13:160. doi: 10.1186/s13578-023-01099-z (PMC10470189; doi:10.1186/s13578-023-01099-z)
Supplement: Supplementary file 6 — Additional File: Table S2 The neutralization potency (IC50) of SARS-CoV-2 mAbs (2–15, XGv347 and LY-CoV1404) [file 13578_2023_1099_MOESM6_ESM.docx]

**Table S2** The neutralization potency (IC_50_) of SARS-CoV-2 mAbs (2-15, XGv347 and LY-CoV1404).

| IC_50_ (ng/ml) | RBD-directed mAbs | | |
| --- | --- | --- | --- |
|  | Class2 | | Class3 |
|  | Infection-elicited | Vaccine-elicited | Infection-elicited |
|  | 2-15 | XGv347 | LY-CoV1404 |
| Wild type | 5 | 9 | 11 |
| BA.1 | >10000 | 6 | 8 |
| R346K | >10000 | - | 2 |
| BA.2 | >10000 | - | 5 |
| BA.4/5 | >10000 | - | 8 |
